# Differential Diagnosis of Neuropsychiatric Presentations: A Combined Assessment of Psychiatric and Neurological Symptoms

**DOI:** 10.1192/j.eurpsy.2025.2033

**Published:** 2025-08-26

**Authors:** F. S. Mir Biribay, M. P. Sanchez Castro, H. Rincon Reques, M. Velasco, C. Serrano, A. Huerta, C. Delicado, M. A. Morillas, M. Bravo

**Affiliations:** 1 Psychiatry; 2 HOSPITAL UNIVERSITARIO LA PAZ, Madrid, Spain

## Abstract

**Introduction:**

Assessing psychiatric and neurological symptoms in emergency settings is crucial for accurate differential diagnosis. Psychosis, seizures, and cognitive impairment often overlap with various medical and psychiatric conditions, making early and precise identification essential for differentiating between potential causes, guiding appropriate treatment, and improving patient outcomes.

**Objectives:**

This report presents a clinical case with initial suspicion of psychotic symptoms accompanied by convulsive features to emphasize the importance of differential diagnosis in neuropsychiatric manifestations.

**Methods:**

This report details a clinical case involving a 26-year-old woman with no prior psychiatric or neurological history, who presented to the emergency department with headaches, insomnia, and unusual behaviors. Three weeks earlier, she had a right parietal focal epileptic seizure and was being treated with lacosamide 100 mg every 12 hours. Post-seizure, she exhibited symptoms such as disorientation, memory lapses, thought blocking, soliloquies, disorganized thinking, and insomnia. Due to suspected psychotic symptoms, the emergency department consulted psychiatry. Both psychiatry and neurology were involved in her evaluation due to the neuropsychiatric nature of her symptoms. The differential diagnosis considered was a first psychotic episode, postictal psychosis, or encephalitis. She was initially prescribed anxiolytics and antipsychotics, including olanzapine 2.5 mg and clonazepam 0.5 mg. Complementary tests, including an EEG, showed right parietal epileptiform discharges and diffuse encephalopathy, but no evidence of status epilepticus. These findings, combined with convulsive symptoms, suggested a neurological origin, leading to her admission to the neurology department.

**Results:**

Further investigation revealed right temporoparietal cortical thickening on brain MRI, indicative of encephalitis, and lumbar puncture results were positive for anti-NMDA receptor antibodies. The patient was then treated with high-dose corticosteroids, later replaced by plasmapheresis every two days. A gynecological consultation was performed to exclude ovarian pathology, and a body CT scan was ordered to rule out tumor-related conditions. Although her condition has stabilized, it is still early to fully assess the effectiveness of the treatment.

**Conclusions:**

This case emphasizes the critical importance of neuropsychiatric differential diagnosis. The overlapping symptoms of psychosis and cognitive impairment can mimic various conditions, making accurate diagnosis challenging. The collaboration between psychiatry and neurology was essential in distinguishing between psychiatric disorders and anti-NMDA receptor encephalitis. Timely and precise diagnosis enabled targeted treatment, demonstrating the need for a multidisciplinary approach in managing complex neuropsychiatric cases.

**Disclosure of Interest:**

None Declared

